# Impact of Chronic HIV Infection on SARS-CoV-2 Infection, COVID-19 Disease and Vaccines

**DOI:** 10.1007/s11904-021-00590-x

**Published:** 2021-11-29

**Authors:** Yexin Yang, Akiko Iwasaki

**Affiliations:** 1 Department of Immunobiology, Yale University School of Medicine, New Haven, CT USA; 2 Department of Epidemiology of Microbial Diseases, Yale School of Public Health, New Haven, CT USA; 3 Howard Hughes Medical Institute, Chevy Chase, MD USA

**Keywords:** SARS-CoV-2 infection, HIV chronic infection, COVID-19, Vaccines

## Abstract

**Purpose of Review:**

The severe acute respiratory syndrome coronavirus 2 (SARS-CoV-2) has developed into a global pandemic that affect the health of hundreds of millions worldwide. In particular, SARS-CoV-2 infection in people with chronic human immune deficiency virus (HIV) infection is of concern, due to their already immunocompromised status. Yet, whether and how the immunological changes brought about by HIV will affect the immune responses against SARS-CoV-2 acute infection and impact the effectiveness of vaccines remain unclear. We discuss the intersection of COVID-19 in HIV-infected individuals.

**Recent Findings:**

People living with HIV (PLWH) may be at increased risk of severe SARS-CoV-2 mediated disease complication due to functional impairment of the immune system and persistent inflammation, which can be ameliorated by antiretroviral therapy. Importantly, limited data suggest that current approved vaccines may be safe and efficacious in PLWH.

**Summary:**

To address remaining questions and supplement limited experimental evidence, more studies examining the interplay between HIV and SARS-CoV-2 through their impact on the host immune system are required.

## Introduction

The emergence of severe acute respiratory syndrome coronavirus 2 (SARS-CoV-2) was first reported in December 2019. Since then, it has put profound stress over the healthcare systems globally and caused over 175 million coronavirus disease 2019 (COVID-19) reported cases with over 3.7 million cumulative deaths as of June 15, 2021 [1]. Vaccines are developed at an unprecedented speed and serve as the key countermeasure against the COVID-19 pandemic. With vaccines being rolled out efficiently, marked declines in the number of weekly reported cases are seen across the globe. While slowing down of the COVID-19 pandemic was reported worldwide, African and South-East Asia regions are suffering from high COVID-19 mortality because of the lack of vaccines, the circulation of new SARS-CoV-2 variants overburdening the healthcare system. Especially, the African region has reported 44% increase in weekly case incidence and 20% increase in new deaths as compared to the previous week [1].

The broad clinical spectrum of COVID-19 was intensively studied soon after the pandemic began. While in most cases, SARS-CoV-2 infection results in mild respiratory symptoms and self-limiting disease, about 15% of patients develop severe clinical manifestations including severe respiratory syndrome and multisystemic failure that require oxygen support and intensive care units (ICU) admission [2–4]. Clinical observations and COVID-19 cohort studies have revealed that age, sex, and body mass index (BMI) are associated with COVID-19 disease course and severity [5–8]. Certain underlying comorbidities, including diabetes, hypertension, cardiovascular disease, and chronic kidney disease, are shown to be associated with severe disease manifestation [5, 6, 9]. Concerns arose in the beginning of the COVID-19 pandemic that immunocompromised patients are at higher risk of more severe SARS-CoV-2 infection because evidence of other respiratory viral infections suggested an association between immunosuppressive condition and high frequencies of superinfection, pneumonia, and death [10, 11]. However, whether compromised immune defense increases the risk of SARS-CoV-2 infection or exacerbates disease progression remains obscure.

African countries are facing a surging wave of COVID-19 pandemics with reporting cases increased for three consecutive weeks [1]. In addition to the COVID-19 pandemic, human immune deficiency virus (HIV) global epidemic has long been a burden of the Sub-Saharan Africa region as it carries 70% of the global HIV infection [12]. Current COVID-19 pandemic has aggravated the situation of people living with HIV (PLWH) worldwide. Medical facilities and physicians previously dedicated for HIV care were partially or fully engaged in fighting the COVID-19 pandemic [13]. In the interim guidance for COVID-19 and persons with HIV issued by the Centers for Disease Control and Prevention (CDC), PLWH are identified to be included in the category of high-risk medical conditions and should weigh the risks and benefits of attending in-person clinical visit related to HIV [14]. Due to shortage of medical resources, quarantine measures, and fears of exposure to SARS-CoV-2, HIV patients were unable to acquire refills of their antiretroviral therapy (ART) medication [15–18]. Several questions regarding the intersection of the rapidly emerged COVID-19 pandemic and long-term HIV epidemic remain to be addressed, which is crucial in mitigating the unintended consequences over PLWH caused by the outbreak of SARS-CoV-2. Here, we review the emerging literature on SARS-CoV-2 infections and some focused studies on SARS-CoV-2 and HIV coinfection and summarize what we know so far about the impact of chronic HIV infection upon SARS-CoV-2 infection and disease outcome. We also review the impact of existing HIV infection status on the COVID-19 vaccine responses. We seek to break down the changes in immunological landscape caused by HIV infection and ART medication and to evaluate the impact of such factors on SARS-CoV-2 infection and pathogenesis.

## Diverse Outcomes in Cohort Studies

Despite the significant heterogeneity and limitation in sample size in case series studies on COVID-19 among solid-organ transplantation (SOT) recipients and people with cancer, many have led to similar conclusions that these patients with immunocompromised condition are at higher risk of developing clinical complication caused by SARS-CoV-2 infection [19••]. Limited case studies of SARS-CoV and Middle East respiratory syndrome (MERS) suggested that PLWH may have lower risk of severe disease progression [20, 21], yet conclusions drawn from focused cohort studies on COVID-19 among PLWH remains controversial.

In a study characterizing 5,700 COVID-19 patients with various predisposed medical conditions, HIV infection accounts for only 0.8% of patients hospitalized with COVID-19 in New York City area, as compared to hypertension and diabetes that represent 56.6% and 33.8% of hospitalized COVID-19 cases in the same cohort [22]. This number was in fact lower than the rate of PLWH in the general population living in the New York City area (1.46%) [23]. Similar results were seen in a cohort study of 20,133 hospitalized COVID-19 patients in the UK [24]. These studies based on large population scale did not reveal a unfavorable effect of HIV infection on COVID-19 disease course. Limitation remains for these studies in that multiple factors that contribute to HIV population heterogeneity including ART adherence, viremia control, and immune reconstitution, were not explicitly examined.

Focused studies comparing COVID-19 disease outcome in patients with or without HIV infection were carried out worldwide. These studies have led to a different conclusion. Comparison analysis performed on samples collected by the International Severe Acute Respiratory and emerging Infections Consortium (ISARIC) World Health Organization (WHO) Clinical Characterization Protocol UK (CCP-UK) study reported an adjusted hazard ratio of 1.69 (95% CI 1.15–2.48; *p* = 0·008) on COVID-19 mortality among PLWH compared to the general population [25]. A higher adjusted hazardous ratio of 2.59 (95% CI 1.74–3.84; *p* < 0·0001) was reported in an analysis based on openSAFELY, the UK primary care database of 1.73 million people and 27.48 thousand HIV infected individuals [26••]. These large databases can achieve great sample size of HIV and SARS-CoV-2 coinfection cases. Nevertheless, neither analysis was able to adjust for confounders including other comorbidities, HIV treatment, and HIV disease progression.

Diverse outcomes are found in other cohort studies focused on studying COVID-19 disease outcome among PLWH, in which patients are further stratified based on therapy, viral burden, immune constitution, and comorbidities. A population cohort study conducted in Sub-Saharan Africa suggested that chronic HIV infection was independently associated with increased COVID-19 mortality similarly across strata of viral load [27••]. In a cohort study on 2,988 HIV and SARS-CoV-2 coinfection cases in New York State, elevated hospitalization and mortality rate were identified in PLWH, and the increase was associated with HIV disease progression stratified by the level of immunosuppression [28]. Yet, another case series of 2,159 hospitalized COVID-19 patients with 31 subjects diagnosed with HIV but were all virologically suppressed on ART showed similar risk of hospitalization among PLWH compared to general population [29].

Whether HIV infected individuals are at higher risk of COVID-19 diagnosis, hospitalization, and mortality remains inconclusive from statistical analysis due to variable results found in the population studies, cohort studies, and case series. It is important to acknowledge that the limitations brought by multiple residual confounders should be taken into consideration when interpreting the results. In addition to rapidly accumulating observational studies reporting the interplay between HIV infection and SARS-CoV-2 pathogenesis based on statistical analysis, comprehensive studies investigating the impact of chronic HIV infection on immune response against SARS-CoV-2 infection experimentally are required to supplement our gap of knowledge.

## Correlates of Immune Protection Against COVID-19

The broad spectrum of COVID-19 disease presentation has been greatly appreciated since the early stage of the current pandemic. Soon after the outbreak began, abundant observational and experimental studies have identified multiple immune biomarkers predicting viral control and disease recovery versus severe disease complications. Our understandings on the immune responses against SARS-CoV-2 infection and immune correlates associated with disease progression have provided insights for development of therapeutic intervention, evaluation of vaccines, and a crucial hint on studying how chronic HIV may affect COVID-19 disease course by modulating the immune system of co-infected patients.

The host immune system is known to be a double-edge-sword in SARS-CoV-2 pathogenesis. On one hand, early evidence suggested that exaggerated host immune response led to increased level of proinflammatory cytokines which induce inflammatory sepsis and acute respiratory distress syndrome (ARDS) [30]. On the other hand, induction of protective immune response leads to control of viral replication and facilitates viral clearance and disease recovery, first suggested by a case study in which the patients with mild symptoms of COVID-19 had normal lymphocyte count, synergetic increase of antibodies and plasma cells, and activation of CD4^+^ and CD8^+^ T cells after onset of symptoms [31].

Lymphopenia was identified as a prominent clinical feature in hospitalized COVID-19 patients. The reduction of blood lymphocyte percentage strongly associates with disease severity and exhibits correlation with disease progression in severely ill patients [32]. The lymphocyte count of CD4^+^ T cells, CD8^+^ T cells, and B cells decreased along with increased severity, suggesting that impaired immune function may play a role in disease progression, while hyperactivated interferon-γ (IFN-γ) producing Th1 cells were increased in severe cases [33]. In a study characterizing SARS-CoV-2 specific T cells, viral spike (S) glycoprotein specific functional CD4^+^ T cells correlate with viral specific IgG and IgA titers [34]. In acute phase of the infection, many of these SARS-CoV-2 specific T cells express activation marker which correlate with early SARS-CoV-2 specific IgG. Viral specific CD8^+^ T cells expressing granzyme B and perforin identified in acute phase were skewed toward memory phenotype in convalescent phase, which are positively associated with disease recovery [35].

Humoral response, as another critical arm of the host adaptive immunity, was successfully targeted by multiple effective vaccines to confer prophylactic intervention of infectious diseases. Most COVID-19 patients develop SARS-CoV-2 specific neutralizing antibodies [36, 37]. Although viral specific antibody titers were found to be elevated in patients with severe disease compared to mild disease, neutralizing antibodies targeting receptor binding domain (RBD) have been shown to be protective rather than being detrimental [37]. In fact, receptor profiling of B cell repertoire in severe COVID-19 patients revealed that extrafollicular responses and clonal expansion of germline clonotypes dominate the COVID-19 antibody secreting cells (ASC) repertoire [38]. While in patients with mild COVID-19, marked increase in the affinity of antibodies targeting prefusion SARS-CoV-2 S protein was observed in a longitudinal study. The affinity maturation of prefusion spike specific antibodies but not S1, S2, or RBD was shown to be associated with disease recovery [39].

More importantly, ample evidence is emerging to indicate that immunological memory is established by SARS-CoV-2 natural infection and vaccination [40–43]. In a study called the SARS-CoV-2 Immunity and Reinfection Evaluation (SIREN) study, 8,278 out of 25,661 enrolled healthcare workers were previously diagnosed with COVID-19 and were seropositive at enrollment. The risk of reinfection of this seropositive group was reduced by 84% compared to the seronegative counterpart [40]. The development of robust neutralizing antibodies is associated with T helper type 1 (Th1) immune response and expansion of RBD specific CD4^+^ and CD8^+^ T cells [42, 44]. These studies collectively emphasized the critical roles of CD4^+^ T cells, CD8^+^ T cells, and B cells in the protective immune response against SARS-Co-2 infection and immunological memory.

## Impact of Chronic HIV Infection on SARS-CoV-2 Susceptibility and COVID-19 Disease Outcome

Despite prevalent concern regarding the risk of SARS-CoV-2 and disease complication in PLWH, the actual impact of chronic HIV infection on COVID-19 is not well characterized. Mixed results were presented by statistical analysis, yet experimental evidence is lacking regarding the mechanism of immune defense against SARS-CoV-2 in PLWH. PLWH are indeed at higher risk of serious influenza-associated complication [45, 46] and bacterial and fungal infection [47, 48], which raises similar concerns in this population at risk of severe COVID-19. By reviewing relevant literature of the immune profile of PLWH and immune correlates of SARS-CoV-2 protection versus immunopathology, we are seeking to learn how HIV chronic infection, HIV associated comorbidities, and ART as prophylactic medication may modulate COVID-19.

### Impact of Immunological Landscape Changes

**Fig. 1:**
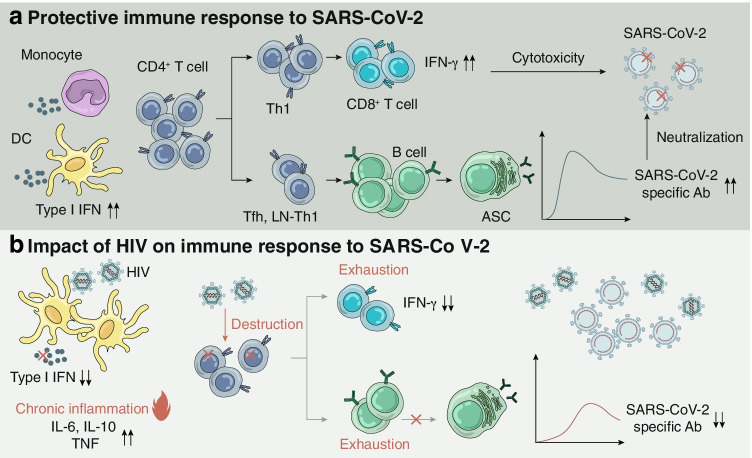
Potential impact of chronic HIV infection on immune response to SARS-CoV-2 infection and immunity. Proposed mechanism of **a** protective immune response to SARS-CoV-2 and **b** potential impact of HIV infection on immune response to SARS-CoV-2. **a** Proper induction of type I IFN stimulate expression of ISGs that mediate antiviral activity in host cells. CD4^+^ T cells are essential in the induction of cellular immune response and humoral immune response, providing CD8^+^ T cells and B cells with help for proper activation and proliferation. IFN-γ secreting CD8^+^ T cells facilitate viral clearance through cell-mediated cytotoxicity. Concurrently, B cell differentiated into ASC which secrete SARS-CoV-2 specific antibodies with neutralizing activity. **b** Chronic HIV infection attenuated type I IFN secretion and lead to chronic inflammation, changing the cytokine milieu that might exacerbate immunopathology. Loss of CD4^+^ T cells, exhaustion of CD8^+^ T cells and B cells occurred in chronic HIV infection, impeding the induction of protective cellular and humoral immune response against SARS-CoV-2 infection. IFN, interferon; ISG, interferon-stimulated genes; Th1, T helper 1 cell; Tfh, T follicular helper cell; LN-Th1, lymph node T helper 1 cell; ASC, antibody secreting cell; SARS-CoV-2, severe acute respiratory syndrome coronavirus 2; Ab, antibody; DC, dendritic cell; HIV, human immune deficiency virus; IL, interleukin; TNF, tumor necrosis factor

People with chronic HIV infection have very distinct immune profiles. In the absence of ART, HIV can establish a progressive infection of human immune cells leading to eventual loss of the CD4^+^ T cells [49] over the course of HIV disease progression (stage 1, CD4^+^ cell count is above 500 cells/μL; stage 2, CD4^+^ cell count is between 350 and 500 cells/μL; stage 3, CD4^+^ cell count is between 200 and 350 cells/μL; stage 4, CD4^+^ cell count is less than 200 cells/μL). Critical loss in CD4 T cell count is a key parameter for defining acquired immunodeficiency syndrome (AIDS). As depletion of CD4^+^ T cells was shown to be associated with worse COVID-19 disease outcome [33], PLWH with low CD4^+^ cell count could have aggravated COVID-19 disease progression due to synergic effect of the two viruses. In a multicenter registry-based cohort reported in the USA, PLWH with CD4^+^ cell count less than 200 cells/μL was associated with increased risk of COVID-19 related mortality and hospitalization compared to those who have higher CD4^+^ cell count [50••]. Although transient lymphopenia is common in many respiratory viral infections [51], SARS-CoV-2 induced lymphopenia appears to be prolonged and more biased towards T cell linage [32, 52, 53]. How exactly low CD4^+^ cell count of people diagnosed with AIDS (stage 4) at onset of COVID-19 disease could contribute to disease progression remains unclear. However, we speculate that progressive loss of CD4^+^ T cells in AIDS patients can potentially impede clearance of SARS-CoV-2 through its effect on antibody production (Fig. 1). One case report of a patient infected with SARS-CoV-2 and HIV whose CD4^+^ cell count was 34 cell/μL showed much delayed SARS-CoV-2 specific IgG and IgM response and prolonged disease course [54]. In a cohort study of 2,017 COVID-19 patients (955 patients with HIV) comparing SARS-CoV-2 specific antibody titers among people with or without HIV, lower anti-RBD IgG (percentage change -53%) and pseudovirus neutralizing antibody titers (percentage change -67%) were observed in HIV infected group (median CD4^+^ cell count = 452 cell/μL) compared to patients without HIV infection [55••]. Depletion of CD4^+^ T cells in a SARS-CoV-2 acute infection model led to diminished antibody response and retarded viral clearance [56••]. Although high-affinity neutralizing antibodies against SARS-CoV-2 can be generated without T follicular helper (T_FH_) cells during infection, lymph node (LN)-Th1 cells was shown to provide complementary help to generate SARS-CoV-2 specific antibodies through interaction with B cells outside of the germinal center (GC) [57].

Additionally, the major target cells of HIV are chemokine receptor (CCR)5 expressing T cells—which represent mucosal memory T cells with preferential expression of CCR5 were greatly targeted for destruction, resulting in a different T cell memory pool in PLWH [58]. Researchers surprisingly found T cells that CD4^+^ memory T cells that cross-react with SARS-CoV-2 in individuals who are naïve to SARS-CoV-2, presumably generated when related human endemic coronavirus were encountered [34]. Whether depletion of pre-existing cross-reactive memory T cells in AIDS patients affect SARS-CoV-2 susceptibility are the remaining important unanswered questions. In fact, cohort studies suggested similar risk of SARS-CoV-2 infection (clinical diagnosis) in PLWH compared to general population, while increased risk of severe disease was reported [28, 59].

Besides progressive depletion of CD4^+^ T cell, chronic HIV infection can also induce qualitative changes in various components of the host immune system. Chronic infection of HIV in which viremia and antigen stimulation persist, CD8^+^ T cells become exhausted [60, 61]. Exhausted CD8^+^ T cells exhibit defects in tumor necrosis factor (TNF) and IFN-γ production, leading to impaired cytotoxic function [62, 63]. Potential exhausted CD8^+^ T cells with expression of inhibitory receptors were found to be associated with severe COVID-19 in early reports [64, 65]. However, these CD8^+^ T cells characterized as terminal exhaustion by expression of inhibitory receptor could be functionally competent for IFN-γ production [66]. Indeed, elevated expression of exhaustion marker could be found on activated CD8^+^ cells and could be beneficial in preventing hyperactivation induced immunopathology, supported by evidence that SARS-CoV-2 specific hyperactivated CD8^+^ T cells in severe illness display impaired exhaustion [67••, 68]. Although the “exhaustion” of CD8^+^ T cells in COVID-19 remains debatable, exhausted lymphocytes in chronic HIV infection were found to be dysfunctional with impaired cytotoxicity [69], which could potentially hamper viral clearance of SARS-CoV-2 during acute infection, when cell mediated immunity coordinate with humoral immunity [56••].

As several reports have shown, marked increases in autoantibodies in severe COVID-19 patients have functional impact over the host immune defense against SARS-CoV-2 and the persistent of inflammation [70–72]. Similarly, these signs of humoral immunity dysregulation are widely appreciated as a hallmark of HIV pathogenesis, characterized by exaggerated B-cell activation and increased production of autoantibodies [73–75]. Another overlapping abnormality of the humoral immune response is the progressive exhaustion of B cells found in both chronic HIV infection [76] and severe COVID-19 disease [77], which could have synergic and undesired effect on antibody-mediated viral clearance and memory establishment.

In addition to changes at the cellular level, HIV infected individuals exhibit local and systemic inflammation; even individuals on ART and viral suppression have chronic inflammation. This likely results from HIV-mediated destruction of mucosal barrier, leading to microbial translocation and dissemination of innate immune stimulus, which was reviewed elsewhere [48, 78]. Of note, inflammatory cytokine milieu characterized in chronic HIV infection [79, 80] shares overlapping targets that are also identified in severe COVID-19 [53, 81], featuring increased level of interleukin-6 (IL-6), IL-10 and tumor necrosis factor (TNF). This argues against the assumption that PLWH might be protected from hyperactivation mediated immunopathology because their immune systems are compromised. On the contrary, a key player of the innate immune system to defend against SARS-CoV-2 acute infection, type I interferons (IFN) [82], is blunted in HIV chronic infection. Several mechanisms by which HIV can suppress the host type I IFN response have been reported. HIV accessory protein Vpr and Vif can target interferon regulatory factors-3 (IRF-3) for degradation [83]. Moreover, HIV gp120 can suppress IFN-α secretion from plasmacytoid dendritic cells, which is the major source of IFN production upon viral recognition [84]. Diminished type I IFN induction is observed in severe COVID-19 cases, and increasing evidence for detrimental effect of blunted IFN response at the onset of disease has been reported in clinical data and mouse model [70, 71, 85].

### Impact of Comorbidities Associated with Chronic HIV

A noteworthy point when discussing COVID-19 disease progression in PLWH is that the clinical spectrum of HIV infection itself is broadly ranging from viral suppression and reconstituted immune system to viremia, immunosuppression, and multiple associated comorbidities. Specific noninfectious comorbidities such as diabetes, cardiovascular disease, and renal failure are significantly more common among PLWH compared to the general population [86••]. Notably, in HIV and SARS-CoV-2 co-infection cases, the most common morbidities are hypertension, diabetes, respiratory disease, liver disease, and renal disease [87]. In a cohort study comparing COVID-19 disease outcome in 22,308 patients with and without HIV (17.8% were HIV infected), a substantial portion of COVID-19 deceased cases in PLWH had diabetes (50%) and hypertension (42%) which were in fact more common in deceased case in patients without HIV (62% for diabetes and 62% for hypertension) [27••]. Therefore, it is possible that the increased risk of hospitalization and worse disease complication may be partially due to a combined effect of HIV-related comorbidities, instead of HIV infection per se. However, HIV infection might contribute to COVID-19 related death that is not associated with such comorbidities.

### Effect of Antiretroviral Therapy

Several case studies have revealed potential beneficial effect of ART on COVID-19 disease outcome among PLWH [88••, 89]. The speculation that ART might act as a pre-exposure prophylactic treatment for SARS-CoV-2 acute infection originated from the fact that several ART drugs have in vitro inhibitory activity against SARS-CoV-2 replication. Tenofovir is a nucleotide analogue that is included in some ART regimen. In vitro molecular docking studies have found that Tenofovir tightly binds SARS-CoV-2 polymerase, suggesting potential effect on blocking viral replication. A multicenter cohort study in Spain reported that tenofovir disoproxil fumarate (TDF)-based ART was associated with lower rate of COVID-19 diagnosis and hospitalization [88••]. However, this association was not found in another cohort study [90]. Another component of ART regimen that is well studied in the setting of SARS-CoV-2 infection is Lopinavir, a protease inhibitor that has been proved to inhibit replication of SARS-CoV [91], MERS-CoV [92], and SARS-CoV-2 [93] in vitro. Yet randomized clinical trial evaluating the effect of lopinavir-ritonavir regimen shows no beneficial outcome in improving clinical outcome or decreasing mortality of COVID-19 [94].

Although the direct effect of ART on restricting SARS-CoV-2 replication remains debatable, it is widely accepted that ART can mitigate HIV-associated immune suppression. Administration of ART can achieve viral suppression and restore CD4^+^ cells count in most HIV infected patients [95]. B cells abnormalities due to persistent antigen stimulation in viremic individuals were restored in the presence of effective ART that decreases HIV viremia [96, 97]. Nevertheless, the persistent hyperactivation of CD8^+^ T cells is still observed in individuals on ART who achieve effective viral suppression but have lower CD4^+^ T cells recovery [98]. Patients on ART could benefit from its effect on restoring a functional immune system rather than by direct suppression of SARS-CoV-2 replication.

## Impact of Chronic HIV Infection on COVID-19 Vaccines

The hope of going back to normal life is brought by the development of several COVID-19 vaccines that achieved profound efficacy and have been rolled out widely in most high-income countries. However, many parts of the world are still suffering from the surging waves of new COVID-19 cases, as a result of vaccines shortage and prevalence of the more transmissible variant strains. With current vaccines production and storage, the allocation of vaccines based on priority is still needed in some developing countries where the pandemic is not under control. In the USA, PLWH was categorized in the group with high-risk health condition by CDC, and according to the guideline, they may receive a COVID-19 vaccine but should be aware of the limited safety data [14]. According to an informal poll from WHO, 24 out of 52 countries have an immunization policy that prioritized PLWH to get the vaccines [99]. However, patients with HIV infection have expressed hesitancy to be immunized with the vaccines that are being rolled out [100, 101]. Here, we discuss what we know so far regarding the safety and efficacy of COVID-19 vaccine among HIV infected individuals.

### Vaccine Safety

Currently approved COVID-19 vaccines are not live attenuated vaccines that can minimally replicate in the recipients. The mRNA vaccines and adenovirus vector-based vaccines all include only the Spike protein gene from SARS-CoV-2 that stimulates antibodies and T cell responses. Vaccine trials from Moderna [102], Pfizer [103••], and Janssen [104] all included patients who were diagnosed with HIV, yet safety data specific for this sub-group have not been published. In a published report of 143 HIV-positive people who received the Pfizer/BioNTech vaccine, the majority of participants have undetectable HIV viral load and an average CD4^+^ cell count of 700 cells/μL, among whom vaccine side effects were reported to be mild [105••]. Another report of 12 people with HIV who received SARS-CoV-2 mRNA vaccines also found only mild side effects [106]. Current studies have not observed higher risk of severe side effects in PLWH, though more focused studies with larger sample size are still required.

Concerns were raised about potential association of HIV acquisition and adenovirus vector-based vaccines [107]. In 2008, a phase II clinical trial of Ad5 vectored HIV vaccines was conducted on 3,000 HIV-1-seronegative participants. Increased risk of HIV infection in Ad5 seropositive men of vaccine group compared to placebo group was reported. Follow-up studies on potential mechanism of Ad5 associated increased susceptibility to HIV infection suggested that pre-existing immunity against the Ad5 vector might facilitate HIV infection and replication in CD4^+^ T cells [108, 109]. Pre-existing immunity against Ad5 characterized by Ad5 neutralizing antibodies was found to be prevalent and of high titers in pediatric and adult population particularly of Sub-Saharan Africa [110]. Therefore, whether the Ad5 vector based CanSino vaccine and the Sputnik V that use Ad5 in the boost dose could result in increased HIV susceptibility may need to be further assessed concerning higher burden of HIV infection in Africa. As HIV infection being the exclusion criteria for clinical trials of these two vaccines [111, 112••], safety evaluation in PLWH were unfortunately not available. The Janssen vaccine uses Ad26 as the adenovirus vector, the pre-existing immunity to which in the population across all regions is markedly lower than Ad5 [110].

### Vaccine Efficacy

The Moderna vaccine trial has recruited 176 people diagnosed with HIV. Zero out of 80 subjects in vaccine group and 1 out of 76 in the placebo group were infected with SARS-CoV-2 during the trial [102]. The Pfizer study recruited 120 HIV-positive participants, who are not included in the phase 2/3 efficacy analysis [103••]. The Janssen studies enrolled 1218 HIV-positive participants. During the follow-up time, there were two COVID-19 cases reported in the vaccine group and four cases reported in the placebo group [104]. Vaccine efficacy was unable to be evaluated according to these trial data due to small number of participants in this sub-group.

Vaccine trials were conducted in South Africa, where B.1.351 variant was dominant, in which PLWH would account for larger percentage of enrolled participants. The Janssen vaccine reported 57% of efficacy in South Africa as compared to 72% in the USA, which might be due to the immune evasion of the B.1.351 variant dominating in South Africa. However, the vaccine is effective in preventing severe cases and death in all regions [113]. Novavax vaccine trial has recruited 2,684 participants (6% of the trial population were HIV-positive) without prior infection with SARS-CoV-2 in South Africa, which showed efficacy of 60.1% among HIV-negative participants and 49.4% efficacy among a mixed group of HIV-positive and HIV-negative participants. Severe COVID-19 cases were not captured in this trial study [114••].

Concerns exist that the dysfunction of the immune system caused by chronic HIV might impair the immune response and establishment of immunological memory after COVID-19 vaccination. The immune protection against secondary SARS-CoV-2 challenge after immunization was found to be largely mediated by humoral immune response but not cellular immunity [56••]. Encouraging result was shown by a study of PLWH who received COVID-19 mRNA vaccine based on small number. Production of SARS-CoV-2 specific antibodies was found in 98% of HIV-positive participants including 12 participants with CD4^+^ cell count less than 350 cell/μl, who are fully vaccinated with the Pfizer/BioNTech vaccine. However, longitudinal study to examine the duration of immunity in this group is required to further evaluate the efficacy of COVID-19 vaccines in PLWH.

## Conclusion

As COVID-19 pandemic still being a huge burden over the healthcare system in Africa, where HIV epidemic has long been a challenge, SARS-CoV-2 and HIV coinfection cases will accumulate. To better protect people suffering from HIV in the current pandemic and potential future threats, we need to continue seeking answers to questions regarding the interaction of these two viruses, which remains largely unaddressed. Current evidence, although inconclusive, suggests that people with chronic HIV infection might be at higher risk of COVID-19 related clinical complication, especially in the setting of viremia and immunosuppression. Though limitations exist in the safety and efficacy evaluation of COVID-19 vaccine in PLWH, weighing risk and benefit, PLWH may still be prioritized in COVID-19 vaccination in the future.
